# Supplementary material for: Inducible auto-phosphorylation regulates a widespread family of nucleotidyltransferase toxins
Source: Nat Commun. 2024 Sep 4;15:7719. doi: 10.1038/s41467-024-51934-1 (PMC11375011; doi:10.1038/s41467-024-51934-1)
Supplement: Supplementary file 1 — Supplementary Information [file 41467_2024_51934_MOESM1_ESM.pdf]

Supplementary Information

Inducible auto-phosphorylation regulates a widespread family of nucleotidyltransferase toxins

Tom J. ARROWSMITH, Xibing XU, Shangze XU, Ben USHER, Peter STOKES, Megan GUEST, Agnieszka K. BRONOWSKA, Pierre GENEVAUX, Tim R. BLOWER.

This file contains nine Supplementary Figures and one Supplementary Table

Supplementary Figure 1. $MenT_1$ is phosphorylated when co-expressed with $MenA_1$ *in vivo*.

Supplementary Figure 2. $MenT_1$ is phosphorylated when incubated with each nucleotide in the presence of $MenA_1$ and $MgCl_2$.

Supplementary Figure 3. D41 and K137 are essential for phosphorylation.

Supplementary Figure 4. Phosphorylation activity is localised to the $MenA_1$ N-terminus.

Supplementary Figure 5. $MenT_1$ is phosphorylated at T39.

Supplementary Figure 6. All four NTPs are viable substrates for phosphorylation activity.

Supplementary Figure 7. Molecular docking predicts a major role of the phosphate tail in nucleotide binding.

Supplementary Figure 8. Phosphorylation blocks heterotrimeric complex formation.

Supplementary Figure 9. MD simulations support the existence of a $MenA_1$:$MenT_1$-p heterodimer in solution.

Supplementary Table 1. Plasmids used in this study.

Supplementary Figures

Supplementary Figure 1

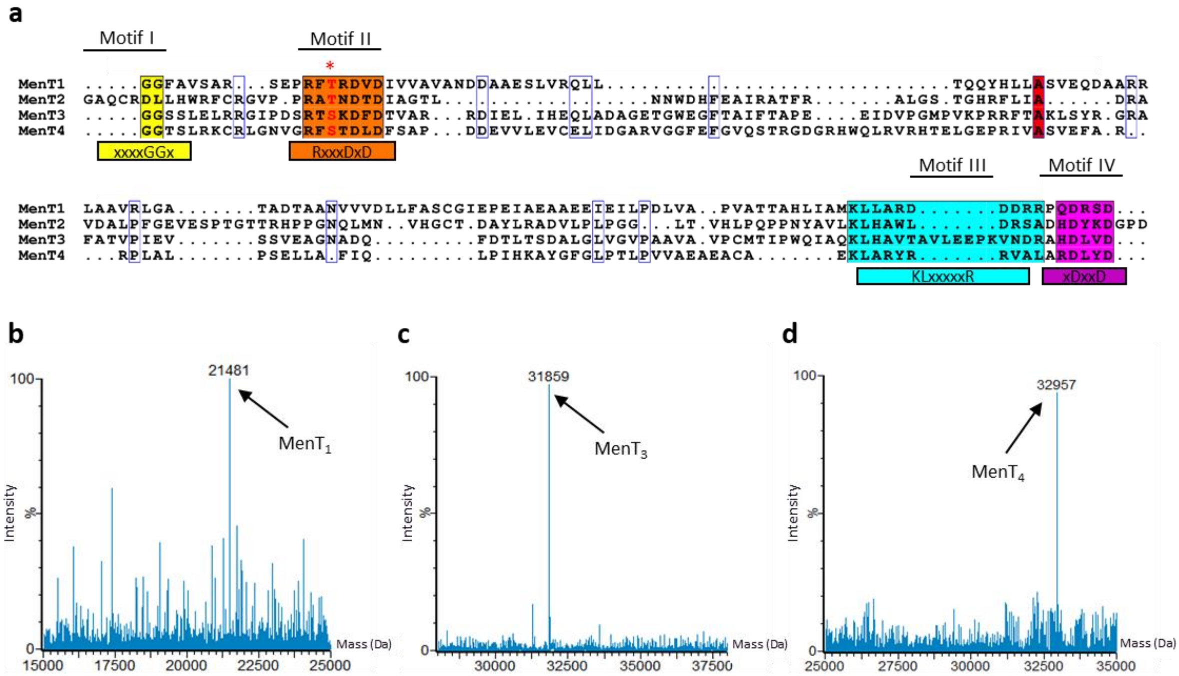

e

Sample	Calculated M_w (kDa)	Observed M_w (kDa)	Observed M_w / Calculated M_w
MenT$_1$	21.5	18.2	0.85
MenT$_1$-MenA$_1$ co-expression	50.4	34.6	0.69

Sample	Calculated R_{st} (Å)	Observed R_{st} (Å)	Observed R_{st}/ Calculated R_{st}
MenT$_1$	22.3	20.2	0.91
MenT$_1$-MenA$_1$ co-expression	30.0	25.8	0.86

f

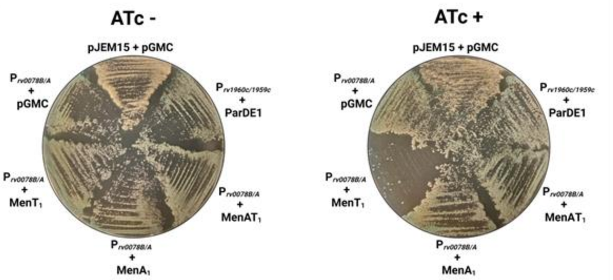

Supplementary Figure 1. MenT$_1$ is phosphorylated when co-expressed with MenA$_1$ *in vivo*. a Sequence alignment of the four MenT toxins from *M. tuberculosis* with conserved DUF1814 motifs coloured yellow (motif I), orange (motif II), cyan (motif III), and purple (motif IV). The site of MenT$_1$ and MenT$_3$ phosphorylation is denoted by a red asterisk. **b-d** ES$^+$-ToF MS of purified MenT$_1$ **(b)**, MenT$_3$ **(c)**, and MenT$_4$ **(d)** expressed in the absence of cognate MenA antitoxins. **e** Observed/calculated Molecular Weight (M_w) and Stokes radii (R_{st}) corresponding to MenT$_1$ expressed in the absence and presence of MenA$_1$. Observed/calculated ratios are coloured green if ≤ 10 %, yellow if > 10 and ≤ 20 %, and red if > 20 % deviation from expected values. Samples were analysed using a HiPrep™ 16/60 Sephacryl® S-200 HR column. **f** Blue/white colony screening of *M. smegmatis* mc^2-155 co-transformed with pJEM15 vector-only, pJEM15-*rv0078B/A* containing a 1000 bp promoter insert (P$_{rv0078B/A}$), or pJEM15-*rv1960c/1959c* containing a 1000 bp promoter insert (P$_{rv1960c/1959c}$), and either pGMC -vector, -MenT$_1$, -MenA$_1$, -MenAT$_1$, or –ParDE1. Screening was performed by plating strains onto LB agar plates supplemented with Km (50 μg.ml^{-1}), Sp (100 μg.ml^{-1}), Tween-80 (0.05% v/v), IPTG (1 mM), and 5-bromo-4-chloro-3-indolyl β-D-galactopyranoside (X-Gal; 40 μg.ml^{-1}), in the presence or absence of the pGMC inducer anhydrotetracycline (ATc; 100 ng.ml^{-1}). Pictures were taken after 4 days incubation at 37 °C. Data are representative of three independent biological replicates.

Supplementary Figure 2

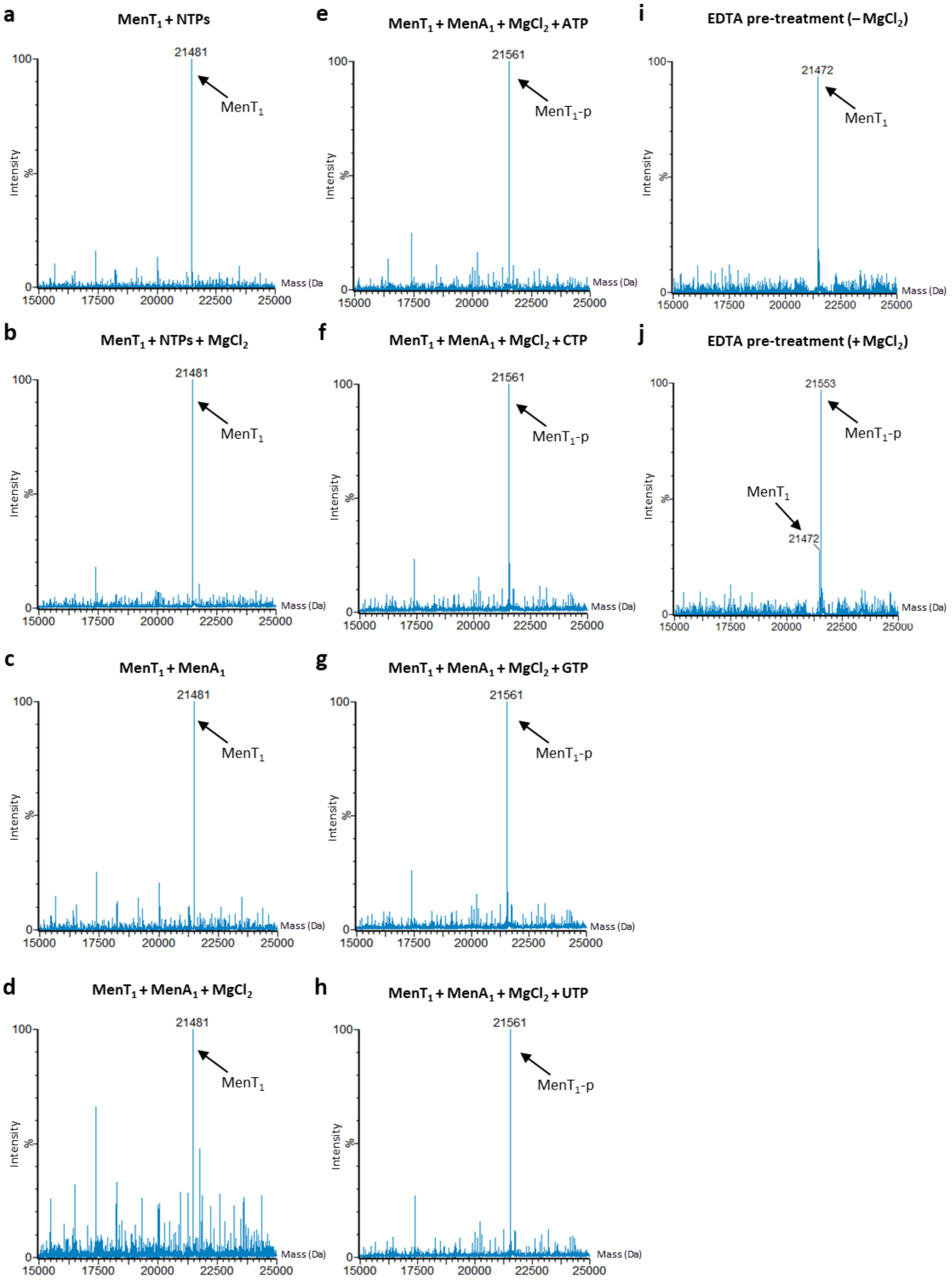

Supplementary Figure 2. $MenT_1$ is phosphorylated when incubated with each nucleotide in the presence of $MenA_1$ and $MgCl_2$. a-h ES^+-ToF MS of $MenT_1$ co-incubated with NTPs in the absence (**a**) or presence (**b**) of $MgCl_2$, $MenT_1$ co-incubated with $MenA_1$ in the absence (**c**) or presence (**d**) of $MgCl_2$, and $MenT_1$ co-incubated with $MenA_1$, $MgCl_2$, and either ATP (**e**), CTP (**f**), GTP (**g**), or UTP (**h**). **i-j** ES^+-ToF MS of $MenT_1$ co-incubated with $MenA_1$ and EDTA for 1 h, prior to the addition of CTP in the absence (**i**) or presence (**j**) of $MgCl_2$. Data are representative of three independent biological replicates.

Supplementary Figure 3

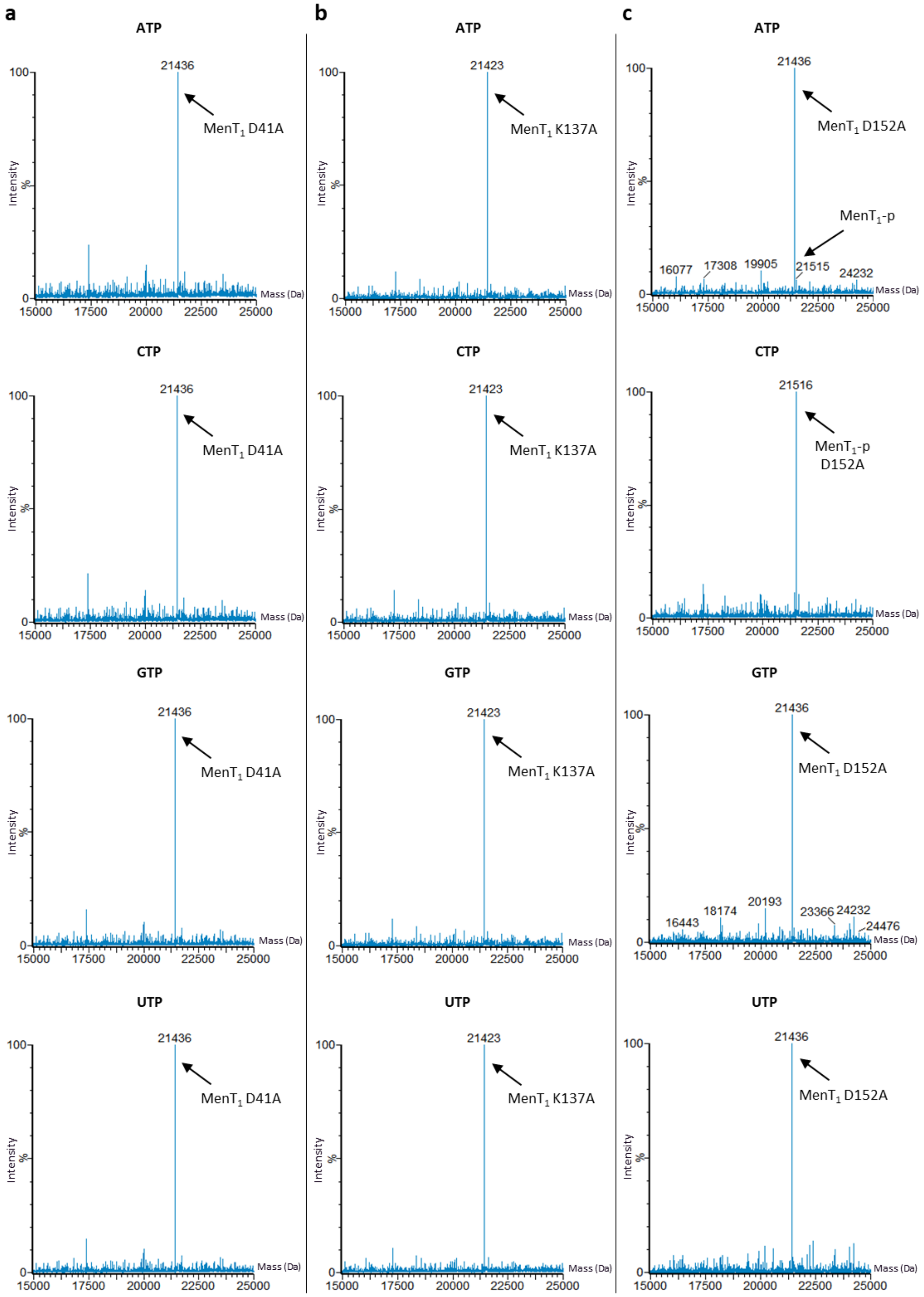

Supplementary Figure 3. D41 and K137 are essential for phosphorylation. a-c ES^+-ToF MS of $MenT_1$ mutants D41A **(a)**, K137A **(b)**, and D152A **(c)** co-incubated with $MgCl_2$, $MenA_1$, and either ATP, CTP, GTP, or UTP. Data are representative of three independent biological replicates.

Supplementary Figure 4

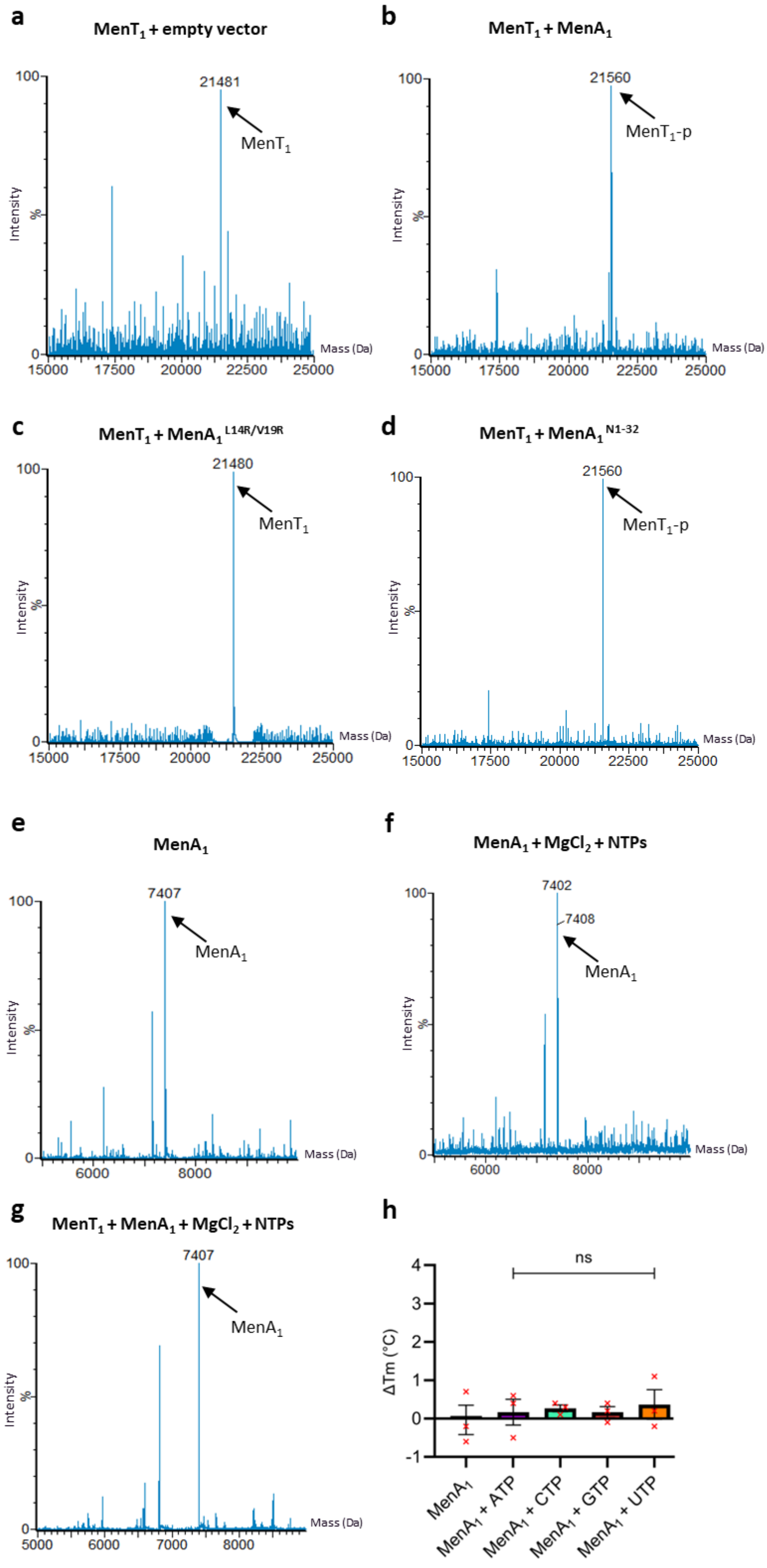

Supplementary Figure 4. Phosphorylation activity is localised to the $MenA_1$ N-terminus. a-d ES^+-ToF MS of purified $MenT_1$ co-expressed with either empty vector (**a**), wild-type $MenA_1$ (**b**), or L14R/V19R (**c**) and N1-32 (**d**) mutants. **e-g** ES^+ ToF MS of purified $MenA_1$ alone (**e**), or $MenA_1$ co-incubated with NTPs and $MgCl_2$, either in the absence (**f**) or presence (**g**) of $MenT_1$. **h** Mean changes in melting temperature following overnight incubation of $MenA_1$ with $MgCl_2$ and either ATP, CTP, GTP, or UTP (one-way ANOVA, p = 0.903). Data are representative of three independent biological replicates and bars display mean values +/- SEM.

Supplementary Figure 5

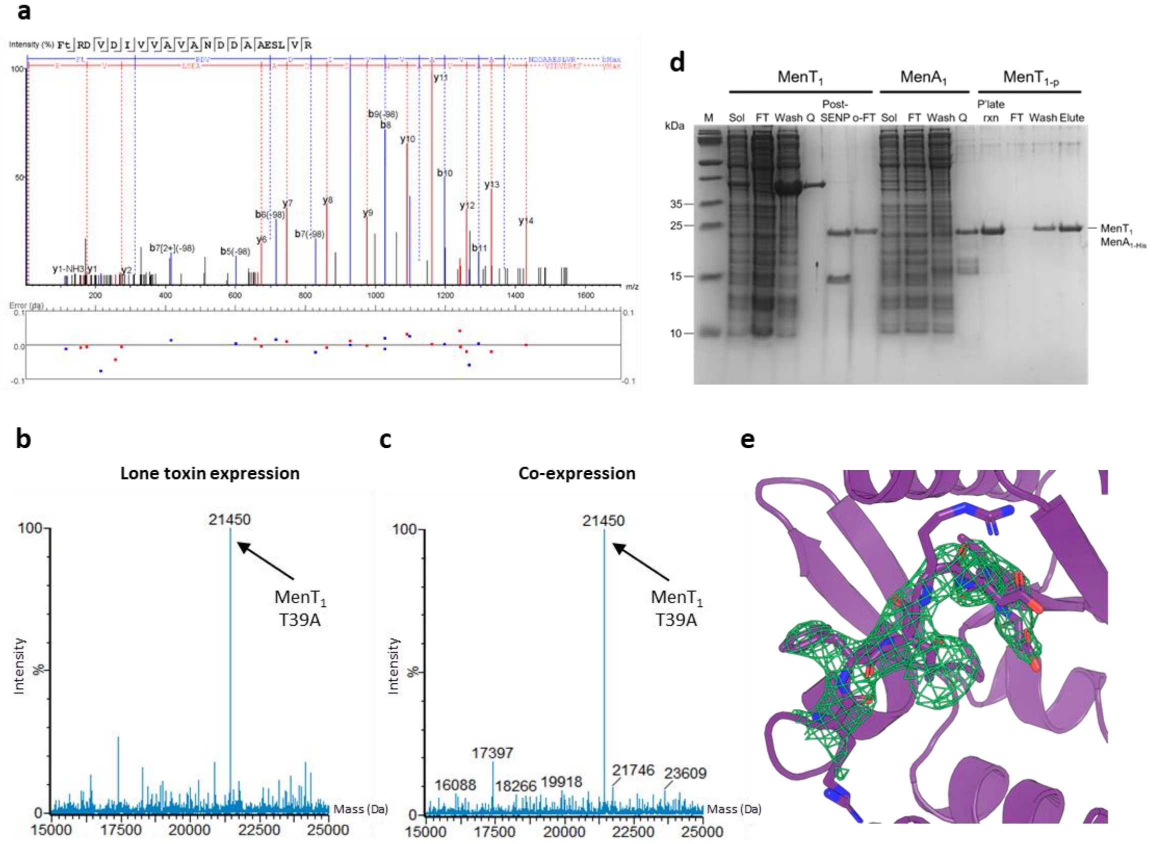

Supplementary Figure 5. MenT$_1$ is phosphorylated at T39. a LC-MS/MS analysis following ProAlanase peptide digests of MenT$_1$ expressed in the presence of MenA$_1$ identified T39 as the site of phosphorylation. Comparison of the masses of two identical fragments ($_{37}$RFTRDVDIVVAVANDDAAESLVRQ$_{60}$) from MenT$_1$ expressed with and without MenA$_1$ revealed the co-expression sample to be 79.966 Da higher than toxin expressed alone, matching the expected mass of a single phosphate. **b-c** ES$^+$-ToF MS of MenT$_1$ T39A expressed and purified in the absence (**c**) and presence (**d**) of MenA$_1$ confirms T39 is the sole site of phosphorylation. Data are representative of three independent biological replicates. **d** SDS-PAGE analysis of MenT$_1$-p fractions during purification. **e** Unbiased F_o-F_c electron density map depicting clear density prior to modelling in of the phosphothreonine.

Supplementary Figure 6

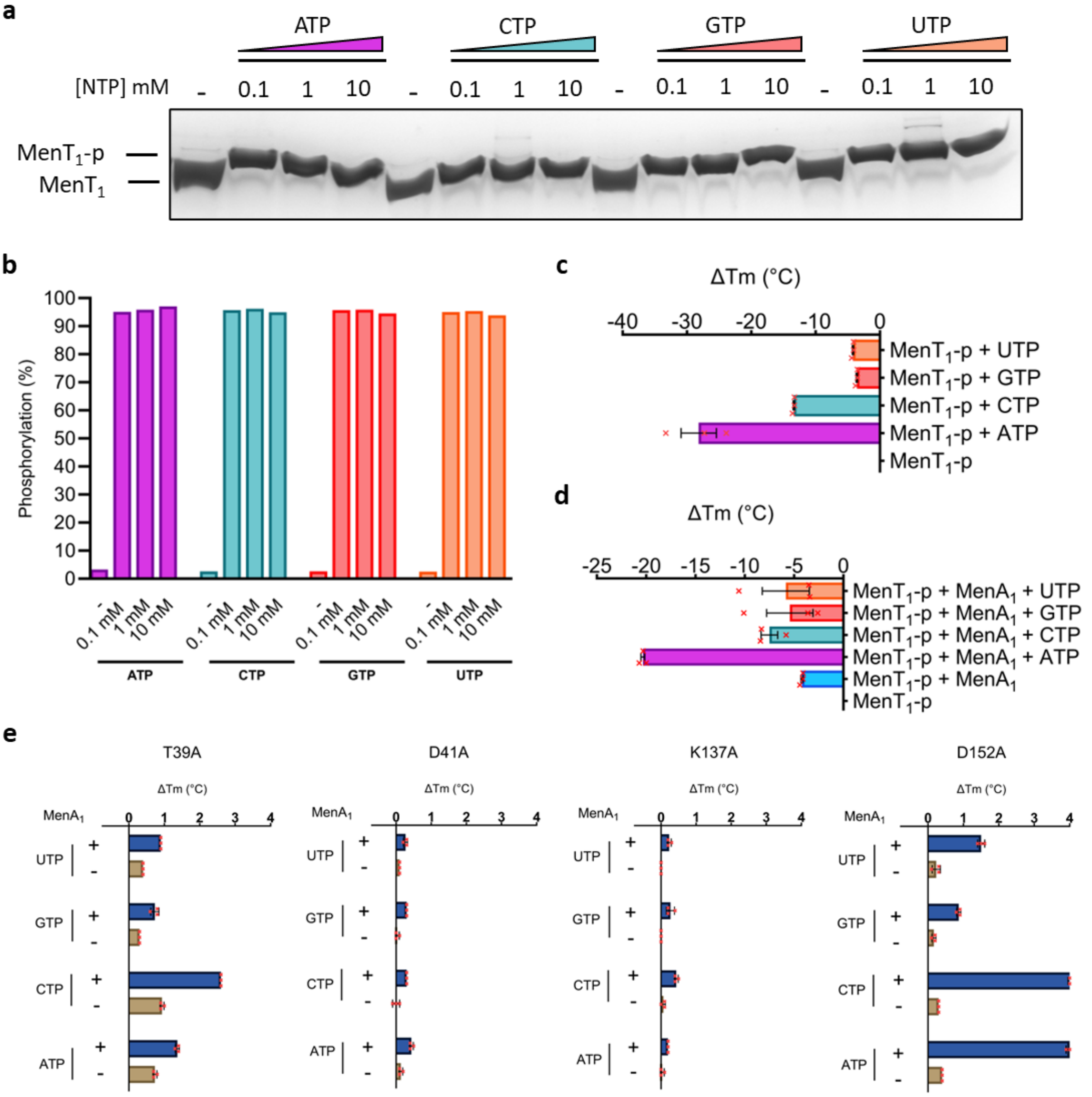

Supplementary Figure 6. All four NTPs are viable substrates for phosphorylation activity. a Phos-Tag SDS-PAGE showing levels of $MenT_1$-p following incubation with $MenA_1$ and $MgCl_2$ in the absence and presence of 0.1 mM, 1 mM, and 10 mM of each NTP. **b** Densitometric analysis of **(a)** reveals a lack of phosphorylation substrate specificity. Data are representative of two independent biological replicates. **c-d** Mean changes in melting temperature following overnight incubation of $MenT_1$-p with $MgCl_2$ and either ATP, CTP, GTP, or UTP, in the absence **(c)** or presence **(d)** of $MenA_1$. **e** Mean changes in melting temperature during thermal shift assays following incubation of $MenT_1$ catalytic mutants T39A, D41A, K137A, or D152A with $MgCl_2$ and ATP, CTP, GTP, or UTP, in the absence or presence of $MenA_1$. Data for panels **c-e** are representative of three independent biological replicates and bars display mean values +/- SEM.

Supplementary Figure 7

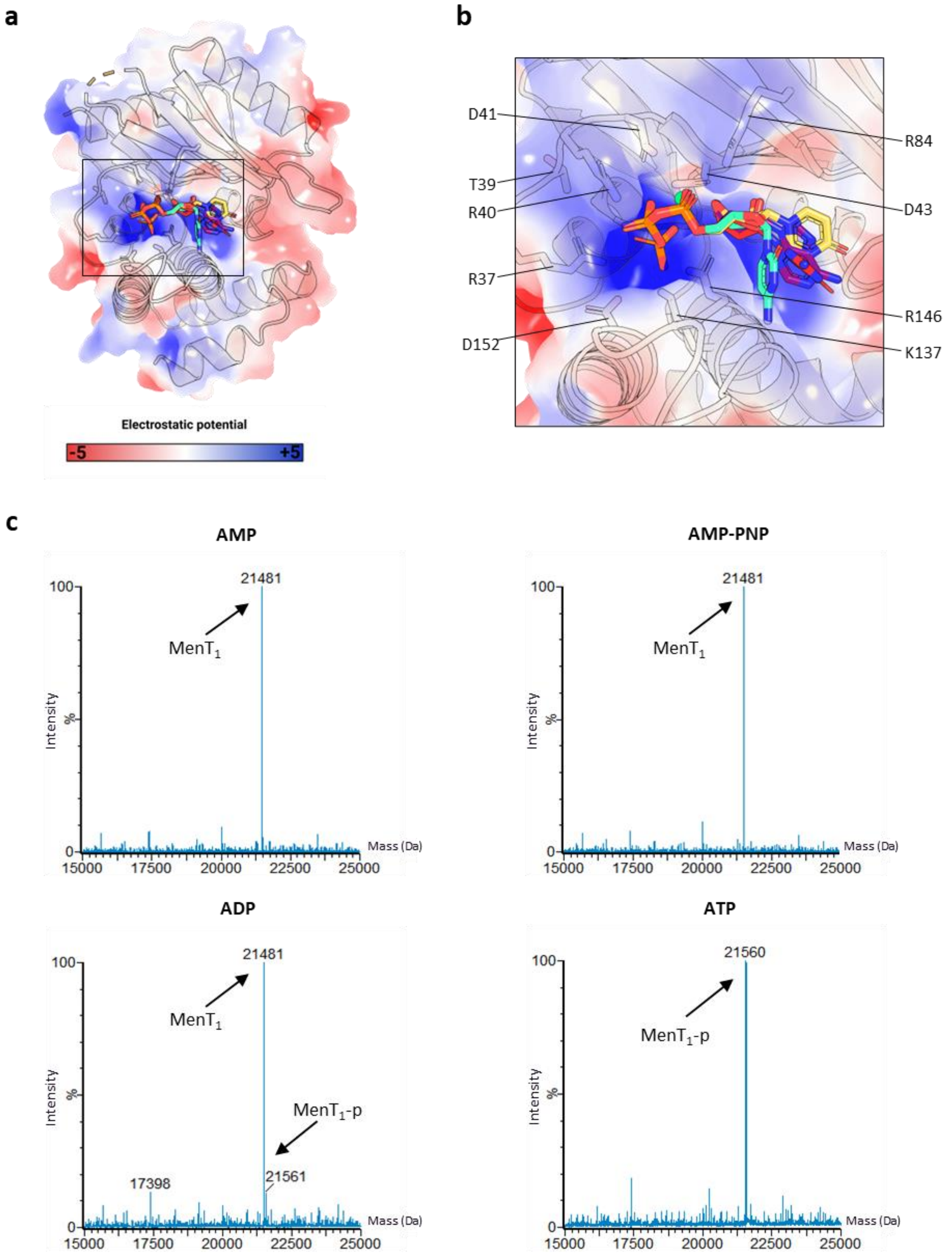

Supplementary Figure 7. Molecular docking predicts a major role of the phosphate tail in nucleotide binding. a Electrostatic surface colouring of $MenT_1$ and structural overlay of best-scored poses for ATP (purple), CTP (teal), GTP (red), and UTP (yellow) following molecular docking of nucleotides and Mg^{2+} to the $MenT_1$ active site. **b** Close-up view as in **(a)**. Residues of interest are shown as sticks, with surface colouring depicting electrostatic potential from -5 $kBTe^{-1}$ (red) to $+5$ $kBTe^{-1}$ (blue), where e is the electron, T is temperature and kB is the Boltzmann constant. Electrostatics were generated using default settings for the APBS plugin (PyMol). **c** ES^+-ToF MS of $MenT_1$ co-incubated with $MgCl_2$, $MenA_1$, and either AMP, ADP, or AMP-PNP. Data are representative of three independent biological replicates.

Supplementary Figure 8

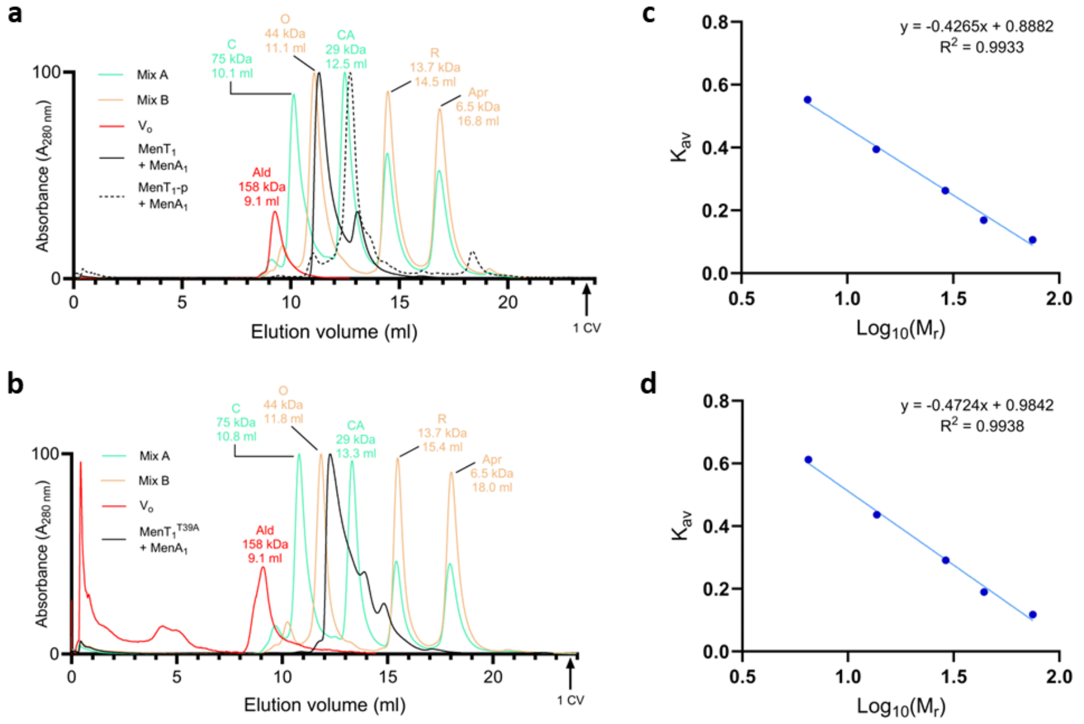

e

	Calculated M_w (kDa)	Observed M_w (kDa)	Observed M_W / Calculated M_W	Calculated R_{st} (Å)	Observed R_{st} (Å)	Observed R_{st}/ Calculated R_{st}
MenT$_1$	21.5	24.9	1.16	22.3	21.2	0.95
MenA$_1$	7.4	17.8	2.41	18.5	19.7	1.06
MenT$_1$:MenA$_1$ heterotrimer	50.4	44.9	0.89	30.0	29.0	0.97

f

	Calculated M_w (kDa)	Observed M_w (kDa)	Observed M_W / Calculated M_W	Calculated R_{st} (Å)	Observed R_{st} (Å)	Observed R_{st}/ Calculated R_{st}
MenT$_1^{T39A}$	21.5	23.4	1.09	22.3	22.0	0.99
MenA$_1$	7.4	12.7	1.72	18.5	17.0	0.92
MenT$_1^{T39A}$:MenA$_1$ heterotrimer	50.3	41.6	0.83	30.0	28.1	0.94

g

	Calculated M_w (kDa)	Observed M_w (kDa)	Observed M_W / Calculated M_W	Calculated R_{st} (Å)	Observed R_{st} (Å)	Observed R_{st}/ Calculated R_{st}
MenT$_1$-p	21.6	20.2	0.94	21.5	20.7	0.96
MenT$_1$-p:MenA$_1$ heterodimer	29.0	26.8	0.92	24.4	23.7	0.97
MenT$_1$-p-MenA$_1$ heterotrimer	50.5	26.8	0.53	29.9	23.7	0.79

Supplementary Figure 8. Phosphorylation blocks heterotrimeric complex formation. a-b Overlaid SEC elution profiles of known molecular weight calibrants against MenT$_1$ + MenA$_1$ and MenT$_1$-p + MenA$_1$ **(a)**, or MenT$_1$ T39A + MenA$_1$ **(b)**. Samples were analysed using a Superdex™ 75 increase 10/300 GL SEC column. Where significant time had elapsed between experiments, the column was re-calibrated. Calibrants; Ald: Aldolase; C: Conalbumin; O: Ovalbumin; CA: Carbonic Anhydrase; R: Ribonuclease A; Apr: Aprotinin. **c-d** Linear plots of K_{av} against Log(M_w) presented with equation of line and R^2 value following column calibration using known molecular weight calibrants; **(c)** corresponds to experiment **(a)**; **(d)** corresponds to experiment **(b)**. **e-g** Observed/calculated Molecular Weight (M_w) and Stokes radii (R_{st}) corresponding to MenA$_1$, and either MenT$_1$ **(e)**, MenT$_1$ T39A **(f)**, or MenT$_1$-p **(g)** incubated in the absence and presence of MenA$_1$. Observed/calculated ratios are coloured green if ≤ 10 %, yellow if > 10 and ≤ 20 %, and red if > 20 % deviation from expected values; **(e)** and **(f)** correspond to experiments **(a)** and **(c)**; **(g)** corresponds to experiments **(b)** and **(d)**.

Supplementary Figure 9

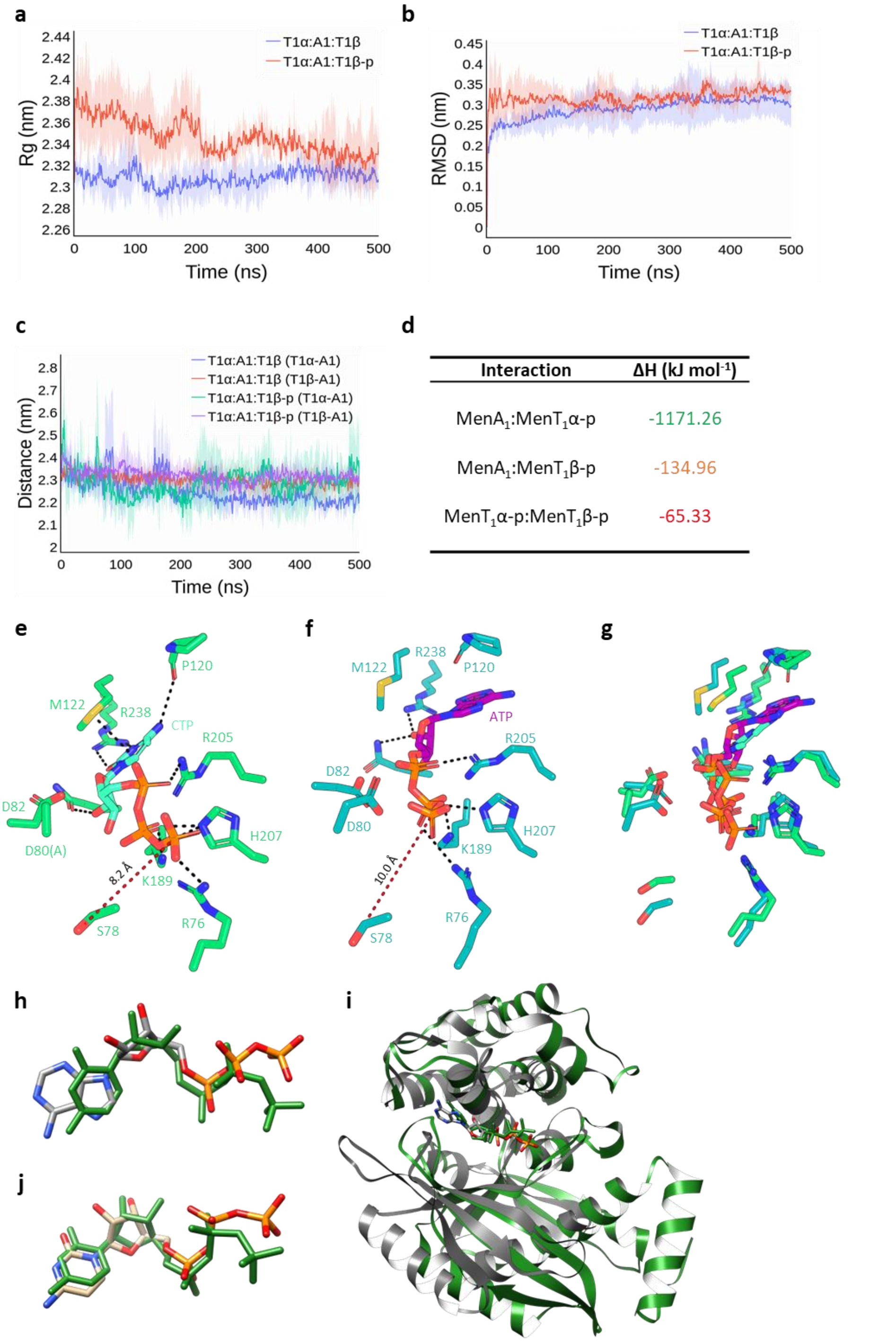

Interaction	ΔH (kJ mol^{-1})
MenA$_1$:MenT$_1$α-p	-1171.26
MenA$_1$:MenT$_1$β-p	-134.96
MenT$_1$α-p:MenT$_1$β-p	-65.33

Supplementary Figure 9. MD simulations support the existence of a MenA$_1$:MenT$_1$-p heterodimer in solution. a Radius of gyration plots for MenT$_1$α:MenA$_1$:MenT$_1$β and MenT$_1$α:MenA$_1$:MenT$_1$β-p during molecular dynamics simulations depicting overall conformational rigidity of non-phosphorylated and mono-phosphorylated trimers. **b** RMSD plots illustrating overall stability of MenT$_1$α:MenA$_1$:MenT$_1$β and MenT$_1$α:MenA$_1$:MenT$_1$β-p trimers during MD simulations. **c** Average distances between respective protomer chains in MenT$_1$α:MenA$_1$:MenT$_1$β and MenT$_1$α:MenA$_1$:MenT$_1$β-p heterotrimer simulations. **d** Protein-protein interaction enthalpies (kJ/mol) calculated for phosphorylated MenT$_1$:MenA$_1$:MenT$_1$ ternary complexes, illustrating stability of the MenA$_1$:MenT$_1$α-p binary complex. **e-f** Close-up binding-site views of the MenT$_3$-CTP crystal structure (**e**; PDB 8XHR) and MenT$_3$ bound to ATP following MD simulations (**f**). Residues involved in nucleotide binding are shown as sticks red for oxygen, blue for nitrogen, and orange for phosphorus, with dashed lines shown to depict protein-ligand interactions. **g** Overlay of poses as in **e-f** depicting high overall similarity between known and predicted binding modes for CTP and ATP respectively. **h** Comparison of the best-scoring pose calculated for ATP bound to MenT$_1$ (colored by element) with the experimental structure of CTP bound to MenT$_3$ (dark green) after superimposing the protein backbones. **i** Overall binding mode of the calculated MenT$_1$–ATP complex (dim gray, ATP colored by element) overlayed onto the MenT$_3$–CTP complex (experimental structure, dark green). **j** The best-scoring pose calculated for CTP binding to MenT$_3$ (CTP colored by element) superimposed onto the experimental structure of CTP binding to MenT$_3$ (dark green). The calculation was performed to validate the molecular docking protocol. Plotted data for **a-c** represent three independent replicates.

Supplementary Table

Supplementary Table 1 - Plasmids used in this study

Gene name	Primers	Primer sequence (5′ to 3′)
pGMC-MenA$_1$T$_1$	Rv0078B In-Fusion For	GAAGACAGGCTGCCCATGGCAGTTTCCGTCGCTGC
	Rv0078A In-Fusion Rev	TGTATAATAAAGTTGTTACCACTTGGCGGCGAGGC
pGMC-MenT$_1$	Rv0078A In-Fusion For	GAAGACAGGCTGCCCATGAACGCTGTGGAGTCGAC
	Rv0078A In-Fusion Rev	TGTATAATAAAGTTGTTACCACTTGGCGGCGAGGC
pGMC-MenA$_1$	Rv0078B In-Fusion For	GAAGACAGGCTGCCCATGGCAGTTTCCGTCGCTGC
	Rv0078B In-Fusion Rev	TGTATAATAAAGTTGTTATGTGAACCGTGTGGACG
pGMC-MenT$_1$ T39A	Rv0078A T39A For	TCCGAACCACGTTTCGCCCGTGACGTGGACATT
	Rv0078A T39C Rev	AATGTCCACGTCACGGGCGAAACGTGGTTCGGA
pGMC-MenT$_1$ T39C	Synthesized and cloned by Genscript	
pGMC-ParDE1	Synthesized and cloned by Genscript	
pJEM15-P$_{rv0078B/A}$	Synthesized and cloned by Genscript	
pJEM15-P$_{rv1960c/1959c}$	Synthesized and cloned by Genscript	
pLAM-MenA$_1$	Rv0078B NdeI For	TTGAATTCCATATGGCAGTTTCCGTCGCTGCGCAG
	Rv0078B EcoRI Rev	TTGAATTCTTATGTGAACCGTGTGGACG
pRARE	Novagen	
pPF656<br>MenA$_3$	pPF1330 Rv1044 For	TTTCATATGCAATTGAGGAGGACAGGGATGTGTGCAAAACCGTATCTAA
	pPF1331 Rv1044 Rev	TTTACTAGTCCCGGGCTTGGTCACGCCGATG
pPF657<br>MenT$_3$	pPF1332 Rv1045 For	TTTCATATGCAATTGAGGAGGACAGGGATGACCAAGCCCTATTCGTC
	pPF1333 Rv1045 Rev	TTTACTAGTCCCGGGTCATCTTTTCGTCGCCC
pPF658<br>MenA$_4$	pPF1334 Rv2827c For	TTTCATATGCAATTGAGGAGGACAGGGATGGT GAGCCCAGCCG
	pPF1335 Rv2827c Rev	TTTACTAGTCCCGGGGTCACGCCTTGCCGATC
pTRB517<br>His$_6$-SUMO-MenT$_3$	TRB1120	TTTGGTACCAAGAAGGAGATATATCCATGAGTGGC
	TRB1121	GCCTCCCGTCTGCTGTTGAA
	TRB1122	TTCAACAGCAGACGGGAGGCACCAAGCCCTATTCGTCGCC
	TRB1124	TTTAAGCTTTTATTATCTTTTCGTCGCCCGATCAA
pTRB544<br>His$_6$-SUMO-MenT$_4$	TRB1175	TTTCCCGGGAAGAAGGAGATATATCCATGAGTGGC
	TRB1176	TTCAACAGCAGACGGGAGGCGCCGGTCTGACCCGTGCG
	TRB1177	TTTAAGCTTTTATTAGGACCGCAGCACCGCCAG
	TRB1121	TTCAACAGCAGACGGGAGGCAACAAAGCTAAATTAACAGC
pTRB597<br>MenA$_1$	TRB1681 Rv0078B For	CAACAGCAGACGGGAGGTAGGAGGACAGGGATGGCAGTTTCCGTCGCTG
	TRB 1682 Rv0078B Rev	GCGAGAACCAAGGAAAGGTTATTATCATGTGAACCGTGTGGACG
pTRB617<br>His$_6$-SUMO-MenA$_1$	TRB1699 Rv0078B For	CAACAGCAGACGGGAGGTGCAGTTTCCGTCGCTGCG
	TRB1700 Rv0078B Rev	GCGAGAACCAAGGAAAGGTTATTATGTGAACCGTGTGG
pTRB629<br>His$_6$-SUMO-MenT$_1$	TRB1701 Rv0078A For	CAACAGCAGACGGGAGGTAACGCTGTGGAGTCGACAC
	TRB1702 Rv0078A Rev	GCGAGAACCAAGGAAAGGTTATTACCACTTGGCGGCGAGGCG
pTRB655<br>His$_6$-SUMO-MenA$_1$ N1-32	TRB1883 For	TAATAACCTTTCCTTGGTTCTCGCATTC
	TRB1884 Rev	CCGTTCACGACCCAGCCT
pTRB669<br>MenA$_3$ D74A	Synthesized and cloned by Genscript	
pTRB670<br>MenA$_3$ S93A	Synthesized and cloned by Genscript	
pTRB671<br>MenA$_3$ H98A	Synthesized and cloned by Genscript	
pTRB672<br>MenA$_3$ D102A	Synthesized and cloned by Genscript	
pTRB673	Synthesized and cloned by Genscript	

$MenA_3$ N104A		
pTRB674<br>$MenA_3$ D155A	Synthesized and cloned by Genscript	
pTRB717<br>$MenA_3$ V103A	Synthesized and cloned by Genscript	
pTRB718<br>$MenA_3$ P105A	Synthesized and cloned by Genscript	
pTRB719<br>$MenA_3$ D102A/N104A	Synthesized and cloned by Genscript	
pTRB720<br>$MenA_3$ D102A/V103A/N104A	Synthesized and cloned by Genscript	
pTRB698<br>His_6-SUMO-$MenT_1$ T39A	Synthesized and cloned by Genscript	
pTRB699<br>His_6-SUMO-$MenT_1$ D41A	Synthesized and cloned by Genscript	
pTRB700<br>His_6-SUMO-$MenT_1$ K137A	Synthesized and cloned by Genscript	
pTRB701<br>His_6-SUMO-$MenT_1$ D152A	Synthesized and cloned by Genscript	
pTRB704<br>His_6-SUMO-$MenA_1$ L14R/V19R	Synthesized and cloned by Genscript	
**tRNA primers**		
M. tuberculosis tRNA Gly-3	Mtb tRNA Gly-3 For	ATTAATACGACTCACTATAGGCGGGCGTAGCTCAATGGT
	Mtb tRNA Gly-3 Rev	TGGAGCGGGCGACGGGAATC
M. tuberculosis tRNA Met-2	Mtb tRNA Met-2 For	ATTAATACGACTCACTATAGGGCGATGTAGCTCAGTCGGTTAGAGCGA
	Mtb tRNA Met-2 Rev	TGGTAGCGATGGCCGGACTCGAA
T7-tRNA	T7-For	ATTAATACGACTCACTAT
	HDV-Rev	AAACGACGGCCAGTGCCAAG
	HDV short-Rev	CTTCTCCCTTAGCCTACCG
M. tuberculosis tRNA T7-Gly3-HDV	Gly3-HDV For	CGATTCCCGTCGCCCGCTCCAGGGTCGGCATGGCATCTC
	Gly3-HDV Rev	GAGATGCCATGCCGACCCTGGAGCGGGCGACGGGAATCG
	T7-Gly3 For	ATTAATACGACTCACTATAGCGGGCGTAGCTCAATGGTA
	HDV-Rev	AAACGACGGCCAGTGCCAAG
	Gly3 Mtb For	GCGGGCGTAGCTCAATGGTAGAGCCCTAGTCTTCCAAACTAGCGACGCGGGTTCGATTCCCGTCGCCCGCTCCA
	HDVFor	GGGTCGGCATGGCATCTCCACCTCCTCGCGGTCCGACCTGGGCTACTTCGGTAGGCTAAGGGAGAAGCTTGGCACTGGCCGTCGTTT
**Sequences of T7-tRNA-HDV constructs**		
T7-Met-2-HDV<br>attaatacgactcactataggcgatgtagctcagtcggttagagcgaacgactcataatcgttaggtcgccggttcgagtccggccatcgctaccagggtcggcatggcatctccacctcctcgcggtccgacctgggctacttcggtaggctaagggagaagcttggcactggccgtcgttt		
