# Supplementary material for: Inducible auto-phosphorylation regulates a widespread family of nucleotidyltransferase toxins
Source: Nat Commun. 2024 Sep 4;15:7719. doi: 10.1038/s41467-024-51934-1 (PMC11375011; doi:10.1038/s41467-024-51934-1)
Supplement: Supplementary file 2 — Reporting Summary [file 41467_2024_51934_MOESM2_ESM.pdf]

nature portfolio

Corresponding author(s): Tim R. Blower

Last updated by author(s): 19th August 2024

Reporting Summary

Nature Portfolio wishes to improve the reproducibility of the work that we publish. This form provides structure for consistency and transparency in reporting. For further information on Nature Portfolio policies, see our Editorial Policies and the Editorial Policy Checklist.

Statistics

For all statistical analyses, confirm that the following items are present in the figure legend, table legend, main text, or Methods section.

n/a	Confirmed	
☐	☒	The exact sample size (*n*) for each experimental group/condition, given as a discrete number and unit of measurement
☐	☒	A statement on whether measurements were taken from distinct samples or whether the same sample was measured repeatedly
☐	☒	The statistical test(s) used AND whether they are one- or two-sided<br>*Only common tests should be described solely by name; describe more complex techniques in the Methods section.*
☒	☐	A description of all covariates tested
☒	☐	A description of any assumptions or corrections, such as tests of normality and adjustment for multiple comparisons
☐	☒	A full description of the statistical parameters including central tendency (e.g. means) or other basic estimates (e.g. regression coefficient) AND variation (e.g. standard deviation) or associated estimates of uncertainty (e.g. confidence intervals)
☐	☒	For null hypothesis testing, the test statistic (e.g. *F*, *t*, *r*) with confidence intervals, effect sizes, degrees of freedom and *P* value noted<br>*Give P values as exact values whenever suitable.*
☒	☐	For Bayesian analysis, information on the choice of priors and Markov chain Monte Carlo settings
☒	☐	For hierarchical and complex designs, identification of the appropriate level for tests and full reporting of outcomes
☒	☐	Estimates of effect sizes (e.g. Cohen's *d*, Pearson's *r*), indicating how they were calculated

Our web collection on statistics for biologists contains articles on many of the points above.

Software and code

Policy information about availability of computer code

Data collection	X-ray data was acquired using Diamond Light Source's "Generic Data Acquisition" (opengda.org) client to run the beamline, and iSpyB (R2.3) to visualise the data. Data were acquired on Diamond Light Source beamlines I04 and I24.
Data analysis	Crystal data analysis:<br>Processing (indexing, integration, scaling) - XDS (Xia2-DIALS) v. Jan 10, 2022<br>Analysis of spacegroups - AIMLESS v8.0.011<br>Molecular replacement - PHASER v8.0.011<br>Initial Building and Refinement - BUCCANEER and REFMAC, respectively, v8.0.011<br>Refinement - PHENIX 1.19.2-4158<br>Building - Coot v0.9.8.7.1<br>Structural figures - PyMol v2.2.2<br><br>Image analysis:<br>Image Lab (Bio-Rad) v6.1<br>ImageJ v1.8.0<br>GraphPad Prism v10.2.0

Mass Spec analysis:
MassLynx v4.1 (Waters)
MaxEnt 3.1

For manuscripts utilizing custom algorithms or software that are central to the research but not yet described in published literature, software must be made available to editors and reviewers. We strongly encourage code deposition in a community repository (e.g. GitHub). See the Nature Portfolio guidelines for submitting code & software for further information.

Data

Policy information about availability of data

All manuscripts must include a data availability statement. This statement should provide the following information, where applicable:

- Accession codes, unique identifiers, or web links for publicly available datasets
- A description of any restrictions on data availability
- For clinical datasets or third party data, please ensure that the statement adheres to our policy

The crystal structures of MenT1-p and MenT3 have been deposited in the Protein Data Bank under accession numbers 8RR5 and 8RR6, respectively. All other data needed to evaluate the conclusions in the paper are present in the paper and/or Supplementary Data.

Research involving human participants, their data, or biological material

Policy information about studies with human participants or human data. See also policy information about sex, gender (identity/presentation), and sexual orientation and race, ethnicity and racism.

Reporting on sex and gender	N/A
Reporting on race, ethnicity, or other socially relevant groupings	N/A
Population characteristics	N/A
Recruitment	N/A
Ethics oversight	N/A

Note that full information on the approval of the study protocol must also be provided in the manuscript.

Field-specific reporting

Please select the one below that is the best fit for your research. If you are not sure, read the appropriate sections before making your selection.

☒ Life sciences ☐ Behavioural & social sciences ☐ Ecological, evolutionary & environmental sciences

For a reference copy of the document with all sections, see nature.com/documents/nr-reporting-summary-flat.pdf

Life sciences study design

All studies must disclose on these points even when the disclosure is negative.

Sample size	No sample-size calculations were performed. For X-ray data, a minimum of 360° datasets were collected for both structures, and then merged with sufficient datasets to optimise resolution as judged within iSpyB and AIMLESS (Diamond Light Source). This provided sufficient data to solve the structures. For other experiments, duplicate or triplicate experiments were performed as standard for the techniques used.
Data exclusions	No data were excluded
Replication	All experimental findings described here were confirmed by repeating the experiments (see figure legends) and, when possible/applicable, by performing distinct experiments to support the same experimental finding. For the structural data, multiple rounds of refinement were performed and they converged to the same structures.
Randomization	Randomisation was not required. There were no covariates applicable within this study and therefore randomization was not needed.
Blinding	Blinding was not required, as it is not necessary for the techniques used.

Reporting for specific materials, systems and methods

We require information from authors about some types of materials, experimental systems and methods used in many studies. Here, indicate whether each material, system or method listed is relevant to your study. If you are not sure if a list item applies to your research, read the appropriate section before selecting a response.

Materials & experimental systems

n/a	Involved in the study
☐	☒ Antibodies
☒	☐ Eukaryotic cell lines
☒	☐ Palaeontology and archaeology
☒	☐ Animals and other organisms
☒	☐ Clinical data
☒	☐ Dual use research of concern
☒	☐ Plants

Methods

n/a	Involved in the study
☒	☐ ChIP-seq
☒	☐ Flow cytometry
☒	☐ MRI-based neuroimaging

Antibodies

Antibodies used	Anti-GFP antibody, Anti-mouse IgG
Validation	Monoclonal anti-GFP (ThermoFisher, Catalog no. MA5-15256) was validated for western blot (https://www.thermofisher.com/antibody/product/GFP-Antibody-clone-GF28R-Monoclonal/MA5-15256). HRP-conjugate anti-mouse IgG (H + L) secondary antibody (Promega W4021) .
