# Supplementary material for: Inducible auto-phosphorylation regulates a widespread family of nucleotidyltransferase toxins
Source: Nat Commun. 2024 Sep 4;15:7719. doi: 10.1038/s41467-024-51934-1 (PMC11375011; doi:10.1038/s41467-024-51934-1)
Supplement: Supplementary file 3 — Supplemenetary Movie 1 [file 41467_2024_51934_MOESM3_ESM.pptx]

## Slide 1
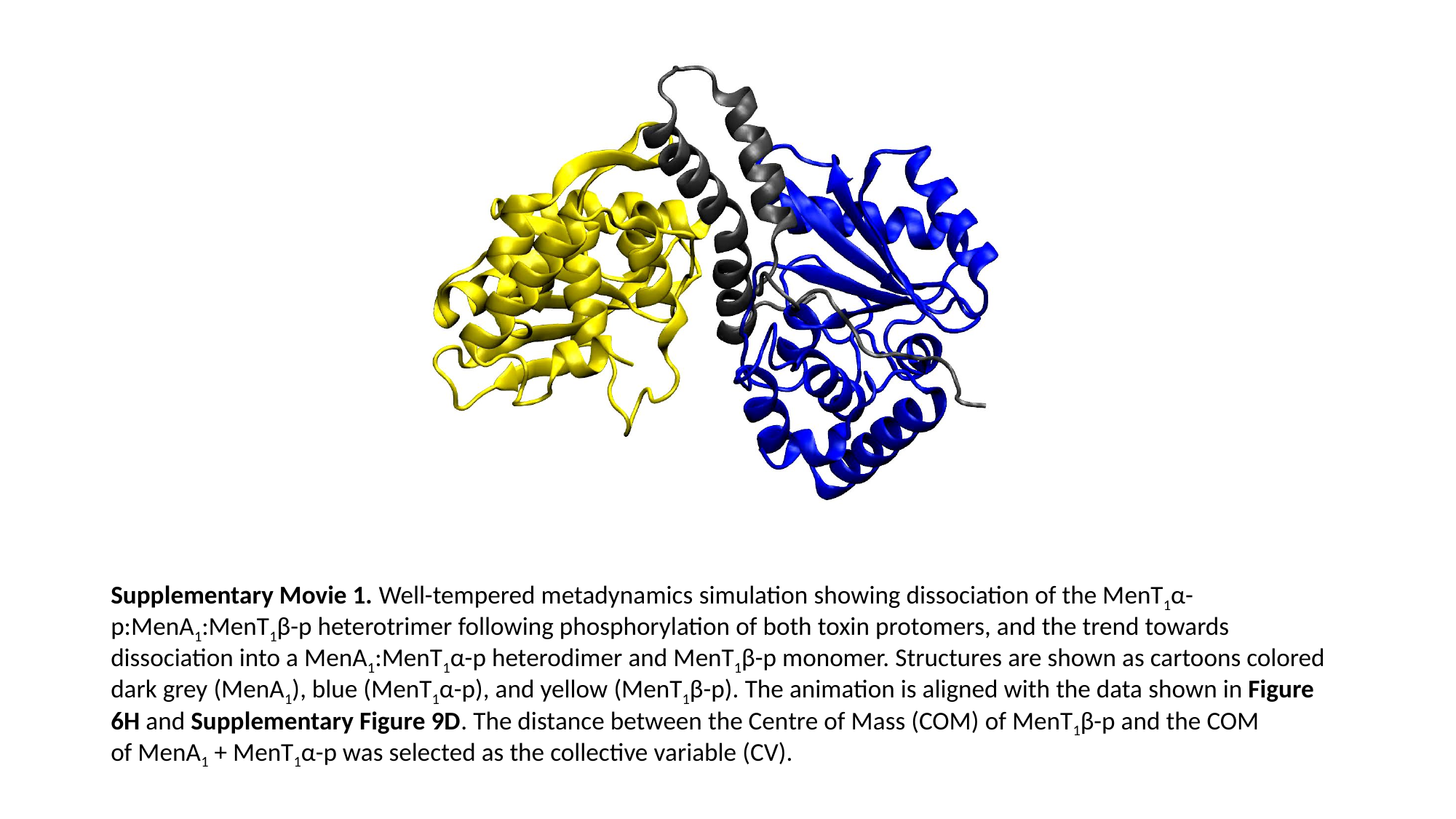

Supplementary Movie 1. Well-tempered metadynamics simulation showing dissociation of the MenT1α-p:MenA1:MenT1β-p heterotrimer following phosphorylation of both toxin protomers, and the trend towards dissociation into a MenA1:MenT1α-p heterodimer and MenT1β-p monomer. Structures are shown as cartoons colored dark grey (MenA1), blue (MenT1α-p), and yellow (MenT1β-p). The animation is aligned with the data shown in Figure 6H and Supplementary Figure 9D. The distance between the Centre of Mass (COM) of MenT1β-p and the COM of MenA1 + MenT1α-p was selected as the collective variable (CV).
